# Continuity of care in general practice and secondary care: retrospective cohort study

**DOI:** 10.3399/BJGP.2024.0579

**Published:** 2025-06-03

**Authors:** Thomas Beaney, Thomas Woodcock, Paul Aylin, Azeem Majeed, Jonathan Clarke

**Affiliations:** The George Institute for Global Health, Imperial College London, London; Department of Primary Care and Public Health, Imperial College London, London; Department of Primary Care and Public Health, Imperial College London, London; Department of Primary Care and Public Health, Imperial College London, London; Centre for Mathematics of Precision Healthcare, Department of Mathematics, Imperial College London, London

**Keywords:** continuity of care, multimorbidity, multiple long-term conditions, primary health care, secondary care, sociodemographic factors

## Abstract

**Background:**

Better continuity in primary and secondary care is linked to improved health outcomes, but it is unclear whether the sociodemographic determinants of continuity are the same in both settings and whether continuity measures in each setting are associated.

**Aim:**

To examine the determinants of relational continuity in general practice and fragmented outpatient specialty care in people with clusters of multiple long-term conditions (LTCs) and the association between continuity in each setting.

**Design and setting:**

A cohort of patients aged ≥18 years registered to general practices in England throughout 2019 and who had linked hospital outpatient records. Patients with ≥2 of 212 LTCs and with ≥3 general practice and ≥3 outpatient appointments were included.

**Method:**

The Continuity of Care Index (COCI) was calculated separately for visits to the same a) GP and b) outpatient specialty, and associations calculated for sociodemographic factors and number of LTCs with COCI scores. The association was also assessed between indices in each setting using univariable and multivariable fractional logit regression.

**Results:**

Of 1 135 903 patients, 56.3% (*n* = 639 489) were aged ≥60 years. Age was the strongest determinant of continuity in general practice, whereas number of LTCs was the strongest determinant in secondary care. Although statistically significant (*P*<0.001), the relationship between the COCI in general practice and outpatients was clinically insignificant in both univariable and multivariable models.

**Conclusion:**

A lack of strong association between continuity of care in general practice and outpatient settings was found. This suggests that fragmented hospital care is not mitigated by increased continuity in general practice.

## Introduction

Relational continuity of care, representing repeated visits to the same doctor,^1^ is associated with better health-related outcomes for patients, including a reduced need for accessing out-of-hours services,^2^ fewer hospital admissions,^3^ reduced rates of inappropriate medication prescribing,^4^ and lower mortality.^5^^,^^6^ People with multiple long-term conditions (MLTCs), defined as having ≥2 long-term conditions (LTCs), often need to see multiple hospital teams to receive the specialist input they require.^7^^,^^8^ Hence, their healthcare interactions are more likely to be ‘fragmented’ across hospital specialties and providers,^9^ creating inefficiencies and corresponding difficulties for patients in navigating health services.^10^^–^12 In this study the authors treated continuity and fragmentation as antonyms.

The importance of continuity to patient care has been shown independently for both primary and secondary care settings, but recently Prior *et al* demonstrated that fragmented care across healthcare settings, including both primary and secondary care, is also associated with inappropriate prescribing of medication and increased mortality.^13^ However, the drivers of fragmented secondary care remain poorly understood, and it is unclear whether it is associated with continuity in general practice. Secondary care outpatient clinics in the UK are typically accessed via electronic referrals from a GP but may also be accessed following an emergency attendance or admission to hospital. General practice has been described as the *‘integrating discipline’*, with unique opportunities to manage the care of people with MLTCs, including both physical and mental health,^14^ and so has a critical role in counter-balancing fragmented hospital care.^8^ Consequently, patients for whom hospital care is most fragmented may be those who have most to gain from relational continuity in general practice. Although sociodemographic determinants of continuity have been identified previously, such as an association of lower GP continuity in some ethnic minority groups and lower continuity with increasing deprivation,^15^ it is unclear whether the patient factors associated with continuity differ between primary and secondary care. It is also unclear whether particular diseases, or clusters of diseases that commonly co-occur in people with MLTCs, are associated with continuity.

The primary aims of this study were to:

- determine the sociodemographic factors associated with relational continuity to the same GP in general practice and continuity of specialties in outpatient care; and
- identify the degree of association between general practice and secondary care continuity.

**How this fits in**

Continuity of care is associated with better patient health outcomes, but it is uncertain whether the determinants of continuity in general practice and secondary care are the same, and whether there is any association between continuity of care in each setting. The determinants of continuity were different at each setting: age was the strongest determinant of general practice continuity, but the number of long-term conditions was the strongest determinant of outpatient continuity. The study demonstrated that there is no meaningful association between relational continuity in general practice and specialty continuity in outpatient care. The findings highlight the different factors explaining continuity in primary versus secondary care and suggest that fragmented hospital care is not mitigated by increased continuity in general practice.

The secondary aims were to identify:

- the associations of individual LTCs with continuity of care; and
- identify whether clusters of diseases that commonly co-occur are at greater risk of low continuity.

## Method

### Data sources

This study used data from the Clinical Practice Research Datalink (CPRD) Aurum, collating patient-level healthcare records from GPs in England.^16^ Secondary care data were sourced from Hospital Episode Statistics (HES), including data on outpatient attendances and hospital admissions. Only patients in CPRD eligible for linkage to HES via a pseudonymised ID were included in the analysis (1.2% of patients were excluded). Data on socioeconomic status were sourced from the 2019 Index of Multiple Deprivation (IMD), which was linked via the lower layer super output area of a person’s residence (the smallest available geographical unit representing between 1000 and 3000 people), where ‘1’ indicates the least deprived decile and ‘10’ the most deprived.^17^

### Cohort inclusion

All patients aged ≥18 years and defined as ‘research acceptable’ by CPRD were eligible.^18^ Those patients registered on 1 January 2019, the study start date, with a full year of follow-up (up to and including 31 December 2019) and registered for ≥1 year before 1 January 2019, were included to allow time for recording of clinical conditions.^19^ To ensure equal follow-up, patients were excluded who had deregistered or died within the follow-up period. Only patients with MLTCs were included, defined as having ≥2 LTCs from a set of 212 defined in earlier work (see Supplementary Box S1).^20^^–^22 To enable calculation of continuity, ≥3 GP and ≥3 outpatient attendances over the year of follow-up were required.^23^ Patient age and gender were used as documented in the CPRD. Ethnicity was categorised into five groups using a code list developed by Davidson *et al* with small modifications.^24^ If missing in the CPRD, the documented ethnicity in HES was used instead (see Supplementary Information S1).

### Continuity measures

The authors of the current study ordered for each patient, separately, their general practice consultations and hospital outpatient appointments chronologically by date during 2019. The GP was identified by a unique identifier given to each member of staff (the ‘staffid’ variable in CPRD; see code categorisation in Supplementary Table S1). Visits to other GP practice staff, such as nurses, were not included. For outpatient appointments, the associated specialty was defined by the treatment function (‘tretspef’) code in HES, which records the specialty of the responsible consultant. Several operational definitions of continuity have been described, a widely used measure of continuity, where ‘0’ represents no continuity and fully fragmented care, and ‘1’ represents all visits to the same GP or specialty (see Supplementary Information S1).^25^^,^^26^ An advantage of this metric over metrics such as the Usual Provider of Care index is that it accounts for the total number of visits.^26^ Therefore, in general practice, continuity is calculated with respect to the clinician a patient sees, whereas in secondary care, continuity is calculated with respect to the clinical specialties attended, over the course of 2019.

### Statistical analysis

Given the distribution of the COCI, constrained to the interval of (0, 1), fractional logit regression was used with robust standard errors calculated.^27^^,^^28^ To analyse patient-level determinants of COCI in general practice and outpatients, sociodemographic factors (age, sex, ethnicity, and IMD decile), number of LTCs, and yearly number of consultations (for both general practice and outpatients) were included as covariates (model 1). Models were tested with and without fixed effects for GP practice and found similar point estimates and confidence intervals (CIs) (compare Supplementary Tables S2 and S3); owing to the computational demand these were not included in further models.

To assess the association between COCI in general practice and outpatients, fractional logit regression was used again and included general practice COCI as the dependent variable and outpatient COCI as the independent variable, categorised into equally spaced quintiles (model 2). This univariable model was compared with two adjusted models: a) a ‘partially adjusted’ model adjusted for sociodemographic factors and number of LTCs; and b) a ‘fully adjusted’ model, additionally adjusted for the total number of GP consultations. All variables were categorised as shown in Table 1. The association of COCI score in general practice was also compared with a) the total number of outpatient appointments, and b) the total number of specialties visited over the year.

**Table 1:** Characteristics of the study population

Patient characteristic	Total, *n* (%), *N* = 1 135 903
**Age, years**	
18–29	67 611 (6.0)
30–39	108 042 (9.5)
40–49	126 316 (11.1)
50–59	194 445 (17.1)
60–69	211 842 (18.6)
70–79	245 434 (21.6)
≥80	182 213 (16.0)

**Gender**	
Female	693 564 (61.1)
Male	442 339 (38.9)

**Ethnicity**	
White	976 609 (86.0)
South Asian	81 594 (7.2)
Black	47 370 (4.2)
Other	15 589 (1.4)
Mixed	12 382 (1.1)
Missing	2359 (0.2)

**IMD decile**	
1 (least deprived)	122 318 (10.8)
2	114 357 (10.1)
3	117 830 (10.4)
4	118 988 (10.5)
5	106 080 (9.3)
6	112 430 (9.9)
7	112 822 (9.9)
8	112 702 (9.9)
9	113 359 (10.0)
10 (most deprived)	104 290 (9.2)
Missing	727 (0.1)

**Number of LTCs**	
2–4	202 530 (17.8)
5–7	255 550 (22.5)
8–10	248 372 (21.9)
11–13	192 852 (17.0)
≥14	236 599 (20.8)

**Number of GP appointments**	
3–4	325 324 (28.6)
5–6	255 855 (22.5)
7–9	250 115 (22.0)
10–14	191 894 (16.9)
≥15	112 715 (9.9)

**Number of outpatient appointments**	
3–4	417 831 (36.8)
5–6	243 759 (21.5)
7–9	201 745 (17.8)
10–14	149 604 (13.2)
≥15	122 964 (10.8)

IMD = Index of Multiple Deprivation.

LTC = long-term condition.

To assess the association of individual LTCs with continuity in each healthcare setting, the presence of an LTC as the independent variable was included, adjusted for sociodemographic factors and number of GP consultations (model 3). Only LTCs occurring in ≥100 people were included (six were excluded, leaving a total of 206 LTCs). Diseases were mapped to 15 corresponding clusters of conditions generated from the current authors’ previous work applied to the same dataset (see Supplementary Box S1).^29^ These data-driven clusters represent diseases that commonly co-occur together and were evaluated by how well they represent clinically established disease associations.^29^ A Kruskal–Wallis test was used to assess statistically significant differences in COCI between clusters. To produce interpretable coefficients for all regression models, the marginal COCI value predicted from the model for each variable is presented, assuming all other covariates were held at their mean values.

### Sensitivity analyses of determinants of continuity

The first sensitivity analysis used the Sequential Continuity Index (SECON) score, an alternative measure that accounts for the order of visits and so gives a longitudinal representation of continuity (see Supplementary Information S1).^25^ The second included only frequent attenders for a) general practice and b) outpatients, defined as those in the top 10% of all patients for total number of yearly GP consultations or outpatient appointments, respectively.

Python (version 3.10.6), Pandas (version 1.4.3), and Stata (version 17.0) were used in analysis.^28^^,^^30^

## Results

### Study participants

Of 6 492 971 patients with MLTCs and aged ≥18 years, registered on 1 January 2019, 1 135 903 (17.5%) with ≥3 GP appointments and ≥3 outpatient appointments in 2019 were included (see Supplementary Figure S1). Older people were more commonly represented than younger people, and there were more females (61.1%) than males (38.9%) (Table 1). Most patients were of White ethnicity, and there were slightly more patients in less deprived IMD deciles (51.1% in the five least deprived deciles). In total, 17.8% of patients had between two and four LTCs, and 20.8% had ≥14. There were 9.9% of patients who had ≥15 GP appointments over the year and 10.8% had ≥15 outpatient appointments.

### Determinants of continuity of care in general practice and outpatients

**Figure 1:**
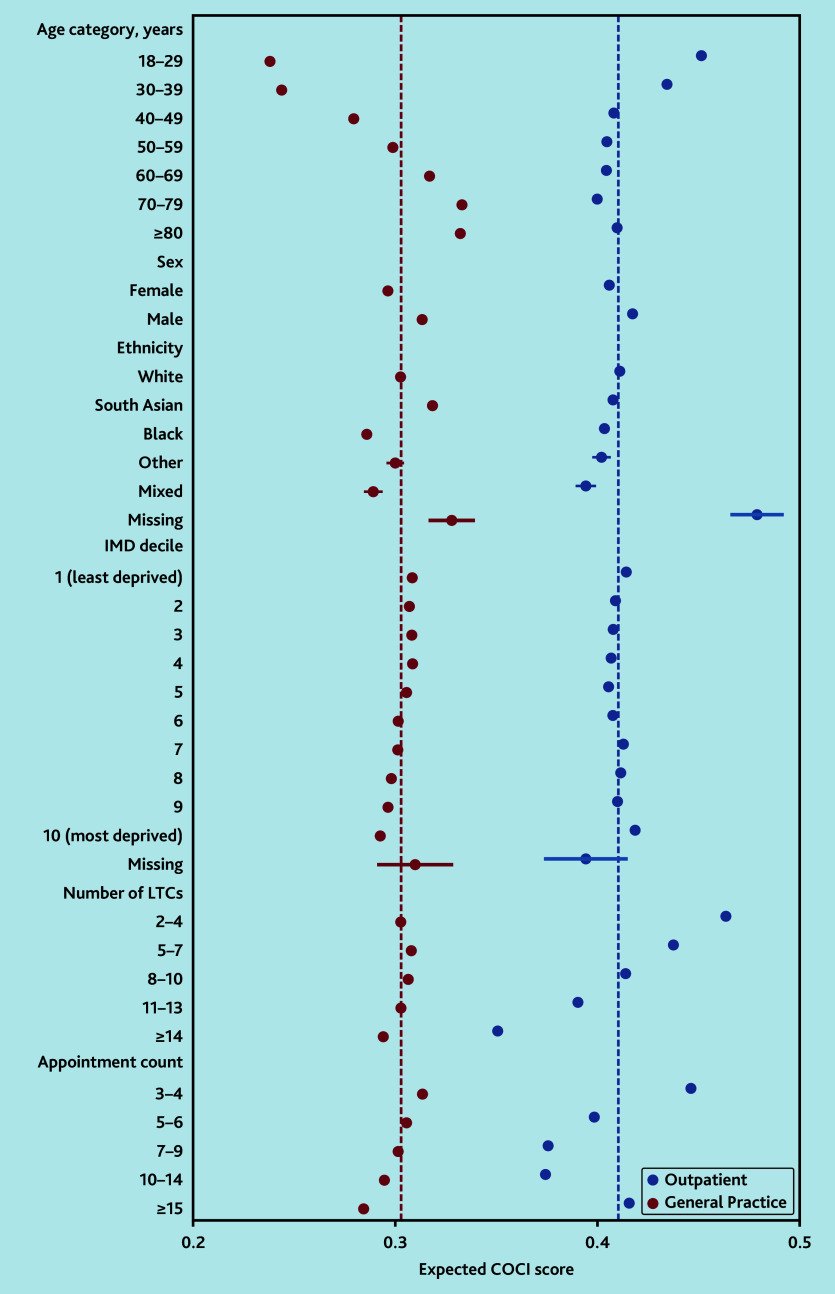
Expected COCI scores by sociodemographic factors for general practice versus hospital outpatients. Predicted values from fractional outcome regression, performed separately for general practice COCI and outpatient COCI, including all covariates in the figure. Appointment count represents total number of either general practice or outpatient appointments. Dots represent expected values and bars represent confidence intervals (exact values given in Supplementary Table S2). Dashed lines represent the expected score for the whole population. COCI = Continuity of Care Index. IMD = Index of Multiple Deprivation. LTC = long-term condition.

Continuity measures in each setting were low. The mean COCI in general practice was 0.304 (standard deviation [SD] 0.268) versus 0.411 (SD 0.291) in outpatient specialties. Age was the strongest sociodemographic determinant of GP continuity, with significantly higher COCI scores on average at older ages, after adjustment for other sociodemographic factors, number of LTCs, and number of GP consultations (predicted COCI of 0.238 [95% CI = 0.236 to 0.240] in those aged 18–29 years versus 0.333 [95% CI = 0.332 to 0.334] in those aged 70–79 years) (model 1). In contrast, within hospital outpatients, the association of COCI with age was smaller and inverted, with higher specialty continuity in those aged <40 years, but similar levels of continuity in those aged ≥40 years. In both settings, continuity was higher in males than females, and lower in Black, Other, and Mixed ethnic groups compared with White (Figure 1 and Supplementary Table S2).

The number of LTCs per person had a strong association with outpatient COCI, with a greater number of LTCs associated with lower continuity. For those with 2–4 LTCs, the predicted COCI was 0.464 (95% CI = 0.462 to 0.465) versus 0.351 (95% CI = 0.350 to 0.352) in those with ≥14 LTCs. In contrast, the number of LTCs had a very small association with GP continuity, after adjustment. Within general practice, higher numbers of consultations were associated with small decreases in the COCI, after adjustment. In outpatients, lower COCI values were expected with an increasing number of appointments up to 14 per year, but in those with ≥15 appointments, the COCI was relatively higher (see Supplementary Table S2).

### Association between general practice and outpatient continuity

There was no correlation between COCI in general practice and outpatients (Pearson correlation coefficient −0.01). In univariable fractional logit regression models (model 2), there were statistically significant (*P*<0.001) but trivial differences in the expected COCI in general practice across quintiles of outpatient COCI in adjusted, partially adjusted, and fully adjusted models (Table 2 and Supplementary Figure S2).

**Table 2:** Expected values of GP COCI by quintiles of outpatient specialty COCI from univariable, partially adjusted and fully adjusted fractional logit regression models

Outpatient COCI	Univariable model, coefficient (95% CI)	Partially adjusted model,^a^ coefficient (95% CI)	Fully adjusted model,^b^ coefficient (95% CI)
**0.00–0.19**	0.307 (0.306 to 0.308)	0.303 (0.302 to 0.304)	0.304 (0.303 to 0.305)
**0.20–0.39**	0.305 (0.305 to 0.306)	0.303 (0.302 to 0.304)	0.303 (0.302 to 0.304)
**0.40–0.59**	0.300 (0.299 to 0.302)	0.300 (0.299 to 0.302)	0.300 (0.299 to 0.301)
**0.60–0.79**	0.299 (0.297 to 0.301)	0.301 (0.299 to 0.303)	0.300 (0.298 to 0.302)
**0.80–1.00**	0.304 (0.302 to 0.305)	0.307 (0.306 to 0.308)	0.304 (0.303 to 0.306)
**Overall**	0.304 (0.304 to 0.305)	0.303 (0.302 to 0.303)	0.303 (0.302 to 0.303)

a Adjusted for sociodemographics.

b Adjusted for sociodemographics and total number of GP consultations.

COCI = Continuity of Care Index.

Associations were also compared between GP COCI and the total number of outpatient appointments and total number of outpatient specialties a patient interacted with over the year but no strong relationships were found (see Supplementary Tables S4 and S5).

### Continuity of care by LTC patterns

**Figure 2:**
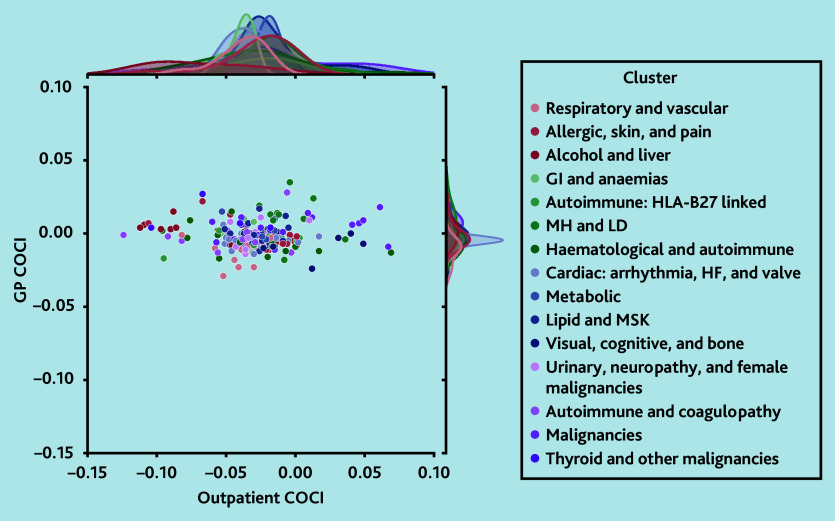
Expected difference in COCI scores for those with each long-term condition (LTC) compared with those without, in general practice versus outpatients, coloured by assigned cluster. The central scatter plot displays each of the 206 LTCs, with the colour representing their assigned cluster (see Supplementary Box S1). The marginal density plots on the top and right sides display the distributions of COCI values for outpatients and general practice, respectively, separately for each cluster. Point estimates for each LTC are given in Supplementary Table S10. COCI = Continuity of Care Index. GI = gastrointestinal. HF = heart failure. HLA = human leukocyte antigen. LD = learning disability. MH = mental health. MSK = musculoskeletal.

There was a much greater spread of expected COCI values in outpatients compared with general practice for 206 individual LTCs after accounting for sociodemographic differences and number of consultations (model 3; Figure 2), but there was no correlation between the two (Pearson correlation coefficient −0.02). Statistically significant differences in COCI values between clusters were found for both general practice and outpatient settings (*P*<0.001 for both), but absolute differences were small (see Supplementary Table S6). Patients with conditions in the ‘alcohol and liver’ cluster showed the largest negative difference in the COCI for outpatient care compared with patients without these conditions (median −0.086, interquartile range [IQR] −0.093 to −0.043), whereas those in the ‘malignancies’ disease cluster had the smallest difference (median −0.015, IQR −0.039 to 0.041).

### Sensitivity analyses

The analyses of determinants of COCI score was repeated in frequent attenders. In general practice the top 10% had ≥17 annual appointments (*n* = 77 283) and in outpatients ≥23 annual appointments (*n* = 43 796). Mean COCI scores were lower in general practice but higher in outpatients (0.282 and 0.449, respectively, compared with 0.304 and 0.411, respectively, in the whole population). Patterns of associations with sociodemographic factors were very similar to the associations for the entire cohort (see Supplementary Figure S3 and Supplementary Table S7). Using SECON, the mean general practice score was 0.362 (SD 0.292) and outpatient score was 0.431 (SD 0.326). Almost identical patterns of associations were again found with sociodemographic factors (see Supplementary Figure S4 and Supplementary Table S8) and a lack of strong association between GP and outpatient scores (see Supplementary Table S9).

## Discussion

### Summary

This study found low relational continuity in general practice and highly fragmented care among hospital outpatient specialties in people with MLTCs, and no meaningful association between relational continuity in general practice and specialty continuity in outpatient care. Despite different interpretations of continuity — in primary care, related to the clinician, and in secondary care, to the specialty — these findings suggest that although patients with MLTCs are most likely to benefit from relational continuity, fragmented hospital care occurs independently of relational continuity with a GP. Furthermore, the patient factors associated with continuity in each setting varied: in general practice, age was the strongest determinant, with older age associated with higher continuity, whereas in outpatient care, a higher number of LTCs was strongly associated with more fragmented care. These associations remained when focusing on patients who were the highest intensity users. Although people with more LTCs will probably interact with more specialties, these results indicate the complex and siloed nature of care for those with MLTCs.

Interestingly, within general practice, individual LTCs and disease clusters explained little of the variability in relational continuity. It is unclear whether a lack of association reflects patient and clinician preferences versus lack of accessibility to consult with the same GP. However, it likely also reflects the limitations of understanding underlying patient needs solely from the presence or absence of a disease. The current research applied an operational definition of continuity suitable for electronic health record data, but this will not capture the nuances of the therapeutic relationship, such as trust between patient and clinician, which may develop over many years.^31^ As others have found, interpersonal continuity of primary care also varies substantially across regions of England, indicating potential for the geography of healthcare provision to influence continuity.^32^

### Strengths and limitations

A strength of the current study is the use of a large and nationally representative sample of patients from CPRD Aurum^16^ along with a large set of 212 LTCs to define the cohort with MLTCs. However, diseases were assumed to be binary states, which will not account for disease severity. Although severity is challenging to establish when using electronic health record data, particularly in the context of a large set of diseases, future work could compare associations with continuity for selected conditions where disease stage can be determined by laboratory values, such as chronic kidney disease. Some LTCs are reviewed more regularly in primary care, for example, those incentivised by the Quality and Outcomes Framework (QOF), which might explain some of the association with continuity. Future work could explore whether LTCs that are reviewed more regularly, or incentivised through QOF, tend to have higher or lower continuity.

The assignment of diseases to clusters was derived from earlier work, based on patterns of co-occurring diseases.^29^ The lack of association between continuity and co-occurrence-based disease clusters may represent a limitation of applying them to understand associations with a future health outcome that was not part of the original clustering process. Indeed, related work exploring the use of the same disease clusters for predicting healthcare utilisation identified greater variability in diseases within clusters than between them, suggesting clusters are less informative of outcomes than are the individual diseases within them.^33^

For secondary care, measures of continuity were calculated between specialties, rather than between clinicians, as a unique identifier for the clinician was not available. Future work could examine clinician-level relational continuity in both settings. Several measures of continuity have been described in the literature.^25^ Although it is uncertain whether some measures perform better in different scenarios, reassuringly, previous research has indicated the similarity of many scores with respect to patient outcomes.^13^ In this study the widely used COCI was applied but similar results were found when SECON was used instead. Continuity over a single calendar year (2019) was investigated in this study, and this was before the COVID-19 pandemic, but future work could explore use of longer time periods and the impact of the pandemic on continuity.

### Comparison with existing literature

An earlier study calculating COCI scores applied to CPRD data reported similar but slightly lower mean values, ranging from 0.21 in the youngest to 0.30 in the oldest age groups.^15^ Stafford *et al*^15^ also found significantly lower continuity in five ethnic minority groups compared with people of White ethnicity, consistent with the results in the current study. In contrast to the findings in the current study, Stafford *et al* found higher continuity scores with an increasing number of LTCs, which may relate to use of a smaller number of LTCs than used in the current study.^15^ To the authors’ knowledge, there are no comparable COCI measures in outpatient care and this is the first study to directly compare measures of continuity in general practice and outpatient settings.

The generalisability of associations between primary and secondary care continuity to other settings may be affected by different healthcare funding models. In England, there are no direct financial incentives or disincentives for a GP to refer a patient for specialist care, although referrals may be restricted and require a patient to meet eligibility requirements. Likewise for patients, there are no direct financial costs of attending NHS outpatient clinics.

### Implications for research and practice

The current findings highlight that although patients with more LTCs may benefit most from greater continuity in GP settings,^8^^,^^14^ they are at higher risk of fragmented outpatient specialty care. Fragmentation of care across hospital specialties may be necessary to meet a patient’s clinical needs, particularly for older people who are more likely to have MLTCs, yet may come at the cost of information continuity and promote siloed management. Although it is uncertain whether the consequences of fragmented secondary care can be offset by relational continuity in primary care, the current study suggests there is no compensatory effect, and that bespoke strategies are needed to promote continuity within each setting and enable integration of care between settings for those with MLTCs.

Increasing GP capacity through recruitment of more GPs, along with changes to funding models to incentivise longer appointment times with the same GP for those with the most complex health problems, would allow GPs to fully assess and manage health needs holistically, which is rarely possible within a 10-minute consultation.^8^ Expanding the use of care navigators for those with MLTCs may also help promote integration between primary and secondary care, but requires evaluation of its effectiveness.^34^ Improving informational continuity, for example, with recently proposed ‘patient passports’ providing a single patient record across the NHS, may also help to mitigate risks associated with frequent transitions of care across healthcare providers.^35^

Better continuity in specialist care could be promoted by greater use of ‘generalist physicians’ and one-stop multispecialty clinics focused on symptoms rather than diagnoses (such as clinics for breathlessness where patients are assessed by respiratory and cardiac teams at the same appointment). These interventions, however, need evaluation to understand their impact on areas such as continuity and quality of care, patient satisfaction, and health outcomes. Future research could also investigate the extent to which continuity in general practice can mitigate the negative impact of fragmented care on health outcomes.

Understanding the determinants of continuity, and how they differ between primary and secondary care, may help to identify groups of patients at highest risk of low continuity, and proactively implement strategies with patients to improve continuity.^36^ However, it is unclear whether quantitative measures of continuity relate to patient perceptions of continuity and patient experience, and there is a need for future research to examine this.

In conclusion, this study demonstrates that there is no meaningful association between relational continuity in general practice and continuity in outpatient specialty care among individuals with MLTCs in England. Importantly, the sociodemographic determinants of continuity varied between settings, with age being the most significant factor in general practice and a higher number of LTCs leading to lower continuity in outpatient care. These findings highlight the fragmented nature of hospital care, which is not mitigated by improved continuity in general practice, underscoring the need for tailored strategies to improve integration across healthcare settings. Addressing the varying determinants of continuity in primary and secondary care will be crucial in designing effective interventions, especially for patients with MLTCs who are most at risk of fragmented care and its adverse consequences.

## Supplementary Information



## Data Availability

This study uses data from CPRD, which are not publicly available to protect patient anonymity. Researchers meeting certain requirements can apply for data access as described here: https://cprd.com/research-applications.
