# Supplementary material for: Continuity of care in general practice and secondary care: retrospective cohort study
Source: Br J Gen Pract. 2025 Jun 3:BJGP.2024.0579. Online ahead of print. doi: 10.3399/BJGP.2024.0579 (PMC12159869; doi:10.3399/BJGP.2024.0579)
Supplement: Supplementary file 1 [file BJGP.2024.0579_suppl.pdf]

Supplementary Information

Continuity of care in general practice and secondary care: retrospective cohort study

Supplementary Box S1. Assignment of 212 LTCs to fifteen disease clusters:

Cardiac: arrhythmia, HF and valve	Malignancies
Atrial Fibrillation<br>Heart failure<br>Nonrheumatic aortic valve disorders<br>Supraventricular tachycardia<br>Nonrheumatic mitral valve disorders<br>Cardiomyopathy: other<br>Pleural effusion<br>Congenital Septal Defect<br>Right bundle branch block combinations<br>Left bundle branch block<br>Dilated cardiomyopathy<br>Hypertrophic Cardiomyopathy<br>Atrioventricular blocks<br>Ventricular tachycardia<br>Rheumatic Valve Disorder<br>Primary pulmonary hypertension<br>Multiple valve disorder<br>Pericardial Effusion<br>Secondary pulmonary hypertension<br>Sick sinus syndrome	Primary Malignancy: Prostate<br>Primary Malignancy: Bowel<br>Primary Malignancy: Lung<br>Secondary Malignancy: other<br>Primary Malignancy: other<br>Primary Malignancy: Oropharyngeal<br>Primary Malignancy: Oesophageal<br>Primary Malignancy: Kidney<br>Primary Malignancy: Pancreas<br>Secondary Malignancy: Liver<br>Primary Malignancy: Stomach<br>Primary Malignancy: Bone<br>Secondary Malignancy: Brain<br>Primary Malignancy: Biliary Tract<br>Secondary Malignancy: Bone<br>Secondary Malignancy: Lung<br>Secondary Malignancy: Lymph Nodes<br>Primary Malignancy: Mesothelioma*<br>Secondary Malignancy: Peritoneum*<br>Secondary Malignancy: Adrenal Gland*<br>Secondary Malignancy: Bowel*<br>Secondary Malignancy: Pleura*<br>Primary Malignancy: Multiple Sites*
**Haematological and autoimmune**	**Allergic, skin and pain**
Bronchiectasis<br>Non-Hodgkin Lymphoma<br>Leukaemia<br>Secondary Thrombocytopaenia<br>HIV<br>Plasma Cell Malignancy<br>Hyposplenism<br>Primary Thrombocytopaenia<br>Myasthenia gravis<br>Hodgkin Lymphoma<br>Sickle Cell Disease<br>Other haemolytic anaemias<br>Myelodysplastic Syndrome<br>Motor neurone disease<br>Hypersplenism<br>Aplastic anaemias<br>Thalassaemia	Asthma<br>Dermatitis<br>Allergic and chronic rhinitis<br>Acne<br>Migraine<br>Irritable bowel syndrome<br>Chronic sinusitis<br>Chronic Fatigue Syndrome<br>Seborrheic dermatitis<br>Dysmenorrhoea<br>Chronic primary pain<br>Endometriosis<br>Polycystic ovarian syndrome<br>Hidradenitis suppurativa<br>Alopecia areata<br>Vitiligo<br>Idiopathic Intracranial Hypertension

Immunodeficiencies	Hypertrophic Nasal Turbinates
**Lipid and MSK**	**Visual, cognitive and bone**
Raised Total Cholesterol<br>Raised LDL-C<br>Osteoarthritis (excl spine)<br>Enthesopathy and synovial disorder<br>Hearing loss<br>Spondylosis<br>Intervertebral disc disorders<br>Polymyalgia Rheumatica<br>Primary Malignancy: Skin<br>Rosacea<br>Tinnitus<br>Parkinson's disease<br>Fibromatosis<br>Spinal stenosis<br>Meniere's Disease<br>Benign essential tremor<br>Spondylolisthesis	Cataract<br>Dementia<br>Osteoporosis<br>Stroke: not otherwise specified<br>Glaucoma<br>Transient ischaemic attack<br>Macular degeneration<br>Visual impairment and blindness<br>Ischaemic stroke<br>Retinal vascular occlusions<br>Giant Cell arteritis<br>Collapsed vertebra<br>Subarachnoid haemorrhage<br>Intracerebral haemorrhage<br>Polycythaemia vera
**MH and LD**	**Respiratory and vascular**
Depression<br>Anxiety disorders<br>Substance Misuse<br>Epilepsy<br>Schizophrenia<br>Intellectual disability<br>Bipolar affective disorder and mania<br>Personality disorders<br>Obsessive-compulsive disorder<br>Hyperkinetic disorders<br>Autism and Asperger's syndrome<br>Eating Disorders<br>Cerebral Palsy<br>Cystic Fibrosis<br>Down's syndrome	COPD<br>Coronary Heart Disease (not otherwise specified)<br>Abdominal Hernia<br>Stable Angina<br>Myocardial Infarction<br>Benign Prostatic Hyperplasia<br>Peripheral Vascular Disease<br>Sleep apnoea<br>Unstable Angina<br>Abdominal Aortic Aneurysm<br>Pleural plaque<br>Asbestosis<br>Secondary polycythaemia
**Metabolic**	**Urinary, neuropathy and female malignancies**
Hypertension<br>Obesity<br>Diabetes Mellitus: other or not specified<br>Type 2 Diabetes Mellitus<br>Raised Triglycerides<br>Low HDL-C	Urinary Incontinence<br>Primary Malignancy: Breast<br>Multiple sclerosis<br>Neuropathic Bladder<br>Trigeminal neuralgia<br>Obstructive and reflux uropathy

Chronic Kidney Disease<br>Gout<br>Diabetic Eye Disease<br>Type 1 Diabetes Mellitus<br>Peripheral Neuropathy<br>Diabetic Neuropathy<br>Autonomic Neuropathy	Primary Malignancy: Ovary<br>Primary Malignancy: Uterus<br>Chronic Cystitis<br>Primary Malignancy: Cervix<br>Spina bifida<br>Endometrial hyperplasia and hypertrophy
**GI and anaemias**	**Autoimmune and coagulopathy**
Vitamin B12 deficiency anaemia<br>Gastro-oesophageal reflux disease<br>Diverticular Disease<br>Gastritis and duodenitis<br>Anaemia: other<br>Diaphragmatic hernia<br>Coeliac disease<br>Barrett's oesophagus<br>Folate deficiency anaemia<br>Primary Malignancy: Bladder<br>Angiodysplasia of colon	Rheumatoid Arthritis<br>Venous thromboembolic disease (Excl PE)<br>Raynaud's syndrome<br>Lupus Erythematosus<br>Glomerulonephritis<br>Hyperparathyroidism<br>Pulmonary Fibrosis<br>Thrombophilia<br>Sjogren's Syndrome<br>Scleroderma<br>Tubulo-interstitial nephritis
**Alcohol and liver**	**Autoimmune: HLA-B27 linked**
Alcohol Misuse<br>Pancreatitis<br>Chronic viral hepatitis<br>Fatty Liver<br>Cirrhosis<br>Alcoholic liver disease<br>Autoimmune liver disease<br>Oesophageal varices<br>Primary Malignancy: Liver<br>Portal hypertension<br>Hepatic failure	Psoriasis<br>Ulcerative colitis<br>Crohn's disease<br>Psoriatic Arthritis<br>Anterior and Intermediate Uveitis<br>Ankylosing spondylitis<br>Sarcoidosis<br>Scleritis and episcleritis<br>Posterior Uveitis<br>Enteropathic arthropathy
**Thyroid and other malignancies**	
Thyroid Disease<br>Primary Malignancy: Melanoma<br>Primary Malignancy: Brain<br>Primary Malignancy: Thyroid<br>Primary Malignancy: Testis	

* Denotes a condition excluded from analyses of individual LTC associations with continuity as <100 individuals with the condition.

Note: Diseases within each cluster ordered by prevalence.

Supplementary Information S1

Method

Ethnicity data was categorised into five groups (Black, Mixed, Other, South Asian, White) based on the latest available ethnicity code recorded in the Clinical Practice Research Datalink (CPRD). We used code lists developed by Davidson *et al* (2021), removing four codes including the term "o/e", representing 'on examination' observed findings rather than self-reported ethnicity.[1] If an ethnicity was recorded as unknown or missing in CPRD, then the most recently recorded value from the Hospital Episode Statistics dataset was used in preference.

The Bice-Boxerman Continuity of Care Index for a patient is given by:

$$\frac{\left(\sum_{i=1}^{P} n_i^2\right) - n}{n(n-1)}$$

where *P* is the total number of specialties (for outpatients) or clinicians (for GP) and *n* is the total number of (outpatient or GP) visits for each patient.

The Sequential Continuity Index (SECON) for a patient is given by:

$$\frac{\sum_{j=1}^{P} s_j}{n-1}$$

and

$$s_j = \begin{cases} 1, & \textit{two consecutive visits to the same speciality/clinician} \\ 0, & \textit{two consecutive visits to different specialties/clinicians} \end{cases}$$

where *P* is the total number of specialties (for outpatients) or clinicians (for GP), *n* is the total number of (outpatient or GP) visits and s_j indicates sequential visits. s_j has a value of 1 if two consecutive visits are to the same specialty (outpatients) or clinician (GP) and 0 otherwise.

Supplementary Table S1. Assignment of staff descriptions (based on CPRD jobcatid) to categories (a value of 1 in each column denotes assignment of this job description to each category)

jobcatid	Description	GP	Nurse	Clinical other	Admin	Other
14	Health Care Support Worker			1		
4	General Medical Practitioner	1				
28	Receptionist				1	
18	Clerical Worker				1	
31	Sessional GP	1				
8	Community Practitioner			1		
1	Consultant			1		
12	Pharmacist			1		
24	GP Registrar	1				
9	Community Nurse		1			
19	Manager				1	
21	System Administrator				1	
25	Medical Student			1		
181	Locum GP	1				
36	Healthcare Assistant			1		
53	Senior Administrator				1	
27	Specialist Nurse Practitioner		1			
7	Midwife			1		
5	Salaried General Practitioner	1				
57	Community Administrator				1	
47	Staff Nurse		1			
30	Medical Secretary				1	
22	Desktop Support Administrator				1	
15	Associate Practitioner - General Practitioner	1				
26	Other Community Health Service - Admin Clerk				1	
34	Physiotherapist			1		
55	Associate Practitioner - Nurse		1			
29	Secretary				1	
71	Dispenser				1	
17	Phlebotomist			1		
16	Counsellor			1		
33	Nurse Consultant		1			
40	Appointments Clerk				1	
37	Medical Technical Officer - Pharmacy			1		
56	Senior Manager				1	
20	Analyst				1	
67	Technician - Admin & Clerical				1	

11	Dietitian			1		
105	Paramedic			1		
78	Paramedic Specialist Practitioner			1		
58	Associate Specialist			1		
52	Community Mental Health Nurse			1		
45	Clinical Coder				1	
61	Sister/Charge Nurse		1			
48	Enrolled Nurse		1			
60	Nurse Manager		1			
83	Other Community Health Service					1
10	Chiropodist/Podiatrist			1		
73	Practitioner			1		
23	System Worker				1	
189	Physician Assistant			1		
74	Information Officer				1	
72	Clinical Assistant			1		
80	Physiotherapist Specialist Practitioner			1		
185	Advanced Practitioner			1		
70	Desktop Support Technician				1	
39	Helpdesk Administrator				1	
51	Helper/Assistant				1	
38	Health Records Administrator				1	
35	Specialist Practitioner			1		
13	Clinical Psychologist			1		
43	Trainee Practitioner			1		
88	Modern Matron		1			
46	Medical Records Clerk				1	
59	Student Practice Nurse		1			
64	Social Care Support Worker					1
32	Clinical Application Administrator				1	
188	Other Community Health Service - Social Care Worker					1
87	Associate Practitioner			1		
95	Assistant Practitioner			1		
84	Call Operator				1	
182	Researcher					1
116	House Officer - Post Registration			1		
183	Assistant GP			1		
107	Availability Monitor					1
62	Psychotherapist			1		
69	Health Records Clerk				1	
106	Physiotherapist Consultant			1		
104	Audit Manager				1	

114	Therapist			1		
42	Social Worker					1
111	Student Nurse - Adult Branch		1			
102	Occupational Therapist			1		
66	Officer					1
6	Midwife - Sister/Charge Nurse			1		
131	Pre-reg Pharmacist			1		
100	Medical Technical Officer			1		
41	Senior House Officer			1		
50	Nursery Nurse					1
118	Dietitian Specialist Practitioner			1		
79	Clinical Team Manager				1	
68	Psychiatrist			1		
109	Gateway Worker					1
85	Community Worker (children)					1
127	Student Community Practitioner					1
75	Network Administrator				1	
81	Helpdesk Technician				1	
110	Medical Records Manager				1	
98	Student Health Visitor			1		
86	Paramedic Consultant			1		
190	Deputising Doctor			1		
112	Audiologist			1		
63	Osteopath			1		
82	Radiographer			1		
142	Student Technician					1
138	Dietitian Consultant			1		
115	Student District Nurse		1			
90	Community Team Manager				1	
101	Midwife - Specialist Practitioner			1		
97	Technician - Additional Clinical Services					1
44	Network Technician				1	
186	Health Visitor			1		
122	Patient Welfare Officer					1
49	Multi Therapist			1		
54	Technician - Healthcare Scientists					1
2	Hospital Practitioner			1		
119	Trust Grade Doctor - SHO level			1		
148	Midwife - Consultant			1		
136	Physiotherapist Manager			1		
140	Social work assistant (mental health)					1
231	Directory of Services Coordinator					1

156	Midwife - Manager			1		
125	Chiropodist/Podiatrist Consultant			1		
132	Mental Health Act Administrator				1	
141	Chiropodist/Podiatrist Specialist Practitioner			1		
137	Healthcare Cadet					1
77	Student Physiotherapist			1		
3	Clinical Medical Officer			1		
89	Asst. Clinical Medical Officer			1		
96	Community Learning Disabilities Nurse			1		
178	Senior Clinical Medical Officer			1		
173	House Officer - Pre-Registration			1		
94	Optometrist			1		
121	Staff Grade			1		
151	Finance Director					1
147	Dietitian Manager			1		
129	Waiting List Clerk				1	
199	Multi Therapist Specialist Practitioner			1		
192	Senior social worker (mental health)					1
159	Biomedical Scientist					1
194	Trainer					1
128	Healthcare Scientist					1
65	Assistant Psychologist			1		
168	Approved Social Worker					1
123	Occupational Therapy Specialist Practitioner			1		
150	Paramedic Manager			1		
91	Specialist Registrar			1		
163	Porter					1
134	Support, Time, Recovery Worker			1		
130	Clinical Director			1		
167	Clinical Director - Medical			1		
92	Chiropodist/Podiatrist Manager			1		
213	Intermediate Care staff					1
207	Healthcare Science Associate					1
154	Radiologist			1		
164	Social services care manager (adults)					1
155	Ward Manager				1	
99	Interpreter					1
158	Radiographer - Diagnostic, Specialist Practitioner			1		
133	Ward Clerk				1	
76	Chaplain					1
113	Radiographer - Diagnostic			1		
160	Board Level Director					1

172	Childcare Co-ordinator					1
126	Trust Grade Doctor - Career Grade level			1		
117	Speech & Language Therapist			1		
157	Waiting List Manager				1	
208	Child Protection worker					1
202	Social work assistant (adults)					1
120	Director of Public Health					1
146	Social services care manager (mental health)					1
219	Social services senior management					1
93	Radiographer - Therapeutic, Manager			1		
212	Occupational Therapist Consultant			1		
176	Medical Director			1		
177	Trust Grade Doctor - Specialist Registrar level			1		
201	Healthcare Science Assistant					1
198	Art Therapist					1
226	Art Therapist Manager					1
145	Radiographer - Diagnostic, Manager			1		
152	Senior social worker (adults)					1
124	Technician - PS&T					1
234	ODP					1
143	Complaints Investigator				1	
165	Student Psychotherapist			1		
222	Nursery manager					1
220	Home Care organiser					1
223	Caldicott Guardian					1
224	Specialist Healthcare Science Practitioner					1
149	Art Therapist Consultant					1
170	Other Executive Director					1
162	Nursing Cadet					1
169	Student Community Mental Health Nurse			1		
153	Student Midwife			1		
135	Art Therapist Specialist Practitioner					1
103	Chief Executive					1
229	Social work team manager (mental health)					1
197	Trust Grade Doctor - House Officer level			1		
218	Occupational Therapist Manager			1		
204	Intermediate Care worker					1
205	Student Occupational Therapist			1		
166	Orthoptist			1		
221	Director of Nursing					1
211	Student School Nurse			1		
217	Social Services information manager					1

215	Art, Music & Drama Student					1
214	Home help					1
171	Student Orthoptist			1		
191	Student Occupational Health Nurse			1		
206	Student Dietitian			1		
203	Social work team manager (adults)					1
175	Outpatient Manager				1	
196	Chair					1
210	General Dental Practitioner			1		
225	Student Nurse - Mental Health Branch			1		
233	OT assistant			1		
232	Student Nurse - Learning Disabilities Branch			1		
193	Regional Dental Officer			1		
230	Playgroup leader					1
195	Cytoscreener					1
228	Assistant Psychotherapist			1		
227	Healthcare Science Practitioner					1
144	Trainee Scientist					1
184	Special salary scale in Public Health Medicine					1
161	Non Executive Director					1
209	Professor					1
180	Reporting Radiographer					1
139	Orthoptist Manager			1		
200	Drama Therapist					1
179	Consultant Healthcare Scientist					1
216	Specialist Healthcare Scientist					1
187	Dental Assistant Clinical Director					1
174	SODP					1
108	Medical Laboratory Assistant					1

Supplementary Table S2. Expected values of GP and outpatient Continuity of Care Index (COCI) for categories of age, sex, ethnicity, deprivation and number of LTCs, from multivariable fractional logit regression models

	GP			Outpatient		
**Variable**	**Expected COCI**	**Lower 95% CI**	**Upper 95% CI**	**Expected COCI**	**Lower 95% CI**	**Upper 95% CI**
**Age category (years)**						
18-29	0.238	0.236	0.240	0.451	0.449	0.454
30-39	0.244	0.242	0.245	0.434	0.432	0.436
40-49	0.279	0.278	0.281	0.408	0.406	0.410
50-59	0.299	0.298	0.300	0.405	0.403	0.406
60-69	0.317	0.316	0.318	0.404	0.403	0.406
70-79	0.333	0.332	0.334	0.400	0.399	0.401
80 or more	0.332	0.331	0.333	0.410	0.408	0.411
**Sex**						
Female	0.296	0.296	0.297	0.406	0.405	0.407
Male	0.313	0.312	0.314	0.417	0.416	0.418
**Ethnicity**						
White	0.303	0.302	0.303	0.411	0.410	0.412
South Asian	0.318	0.317	0.320	0.408	0.406	0.410
Black	0.286	0.284	0.288	0.403	0.401	0.406
Other	0.300	0.296	0.304	0.402	0.397	0.407
Mixed	0.289	0.284	0.294	0.394	0.389	0.399
Missing	0.328	0.316	0.339	0.479	0.466	0.492
**Index of Multiple Deprivation decile**						
1 (least deprived)	0.308	0.307	0.310	0.414	0.413	0.416
2	0.307	0.305	0.309	0.409	0.407	0.411
3	0.308	0.307	0.310	0.408	0.406	0.409
4	0.309	0.307	0.310	0.407	0.405	0.408
5	0.306	0.304	0.307	0.405	0.404	0.407
6	0.301	0.300	0.303	0.408	0.406	0.409
7	0.301	0.300	0.303	0.413	0.411	0.415
8	0.298	0.296	0.300	0.411	0.410	0.413
9	0.296	0.295	0.298	0.410	0.408	0.412
10 (most deprived)	0.293	0.291	0.294	0.419	0.417	0.420
Missing	0.310	0.291	0.329	0.394	0.374	0.415
**Number of LTCs**						
2-4	0.303	0.301	0.304	0.464	0.462	0.465
5-7	0.308	0.307	0.309	0.438	0.436	0.439
8-10	0.306	0.305	0.307	0.414	0.413	0.415
11-13	0.303	0.302	0.304	0.390	0.389	0.392
14 or more	0.294	0.293	0.295	0.351	0.350	0.352
**Appointment count**						
3-4	0.313	0.312	0.315	0.446	0.445	0.447
5-6	0.306	0.305	0.307	0.398	0.397	0.400
7-9	0.301	0.300	0.302	0.376	0.375	0.377
10-14	0.295	0.294	0.296	0.374	0.373	0.375
15 or more	0.284	0.283	0.286	0.416	0.414	0.417
**Overall**	**0.303**	**0.302**	**0.303**	**0.410**	**0.410**	**0.411**

Note: Models adjusted for all variables shown in the table. Estimates in Figure 1 present the difference between the overall population score and COCI scores for each variable.

Supplementary Table S3. Expected values of GP and outpatient Continuity of Care Index (COCI) for categories of age, sex, ethnicity, deprivation and number of LTCs, from multivariable fractional logit regression models, including practice fixed effects

	GP			Outpatient		
**Variable**	**Expected COCI**	**Lower 95% CI**	**Upper 95% CI**	**Expected COCI**	**Lower 95% CI**	**Upper 95% CI**
**Age category (years)**						
18-29	0.239	0.237	0.240	0.445	0.442	0.447
30-39	0.247	0.246	0.249	0.429	0.427	0.431
40-49	0.283	0.281	0.284	0.406	0.405	0.408
50-59	0.301	0.299	0.302	0.404	0.403	0.406
60-69	0.316	0.315	0.318	0.405	0.403	0.406
70-79	0.330	0.329	0.331	0.400	0.399	0.401
80 or more	0.328	0.327	0.329	0.411	0.410	0.413
**Sex**						
Female	0.296	0.296	0.297	0.405	0.405	0.406
Male	0.312	0.312	0.313	0.416	0.415	0.417
**Ethnicity**						
White	0.303	0.302	0.303	0.408	0.408	0.409
South Asian	0.313	0.311	0.315	0.413	0.411	0.415
Black	0.290	0.287	0.293	0.408	0.405	0.411
Other	0.303	0.298	0.307	0.406	0.401	0.410
Mixed	0.292	0.288	0.297	0.401	0.396	0.406
Missing	0.330	0.319	0.341	0.474	0.462	0.486
**Index of Multiple Deprivation decile**						
1 (least deprived)	0.312	0.310	0.314	0.411	0.409	0.413
2	0.309	0.308	0.311	0.411	0.410	0.413
3	0.307	0.305	0.308	0.410	0.409	0.412
4	0.307	0.306	0.309	0.408	0.406	0.409
5	0.305	0.304	0.307	0.409	0.407	0.411
6	0.301	0.299	0.302	0.410	0.408	0.412
7	0.299	0.298	0.301	0.409	0.408	0.411
8	0.299	0.297	0.300	0.409	0.407	0.411
9	0.295	0.293	0.296	0.409	0.407	0.411
10 (most deprived)	0.292	0.290	0.294	0.413	0.411	0.415
Missing	0.312	0.293	0.331	0.405	0.383	0.427
**Number of LTCs**						
2-4	0.299	0.298	0.301	0.468	0.466	0.469
5-7	0.305	0.304	0.306	0.440	0.438	0.441
8-10	0.305	0.304	0.306	0.414	0.412	0.415
11-13	0.304	0.303	0.305	0.388	0.386	0.389
14 or more	0.296	0.295	0.297	0.346	0.345	0.347
**Appointment count**						
3-4	0.311	0.310	0.312	0.443	0.442	0.444
5-6	0.304	0.303	0.304	0.397	0.396	0.398
7-9	0.300	0.299	0.301	0.376	0.375	0.377
10-14	0.296	0.295	0.297	0.376	0.375	0.377
15 or more	0.287	0.286	0.288	0.420	0.418	0.421
**Overall**	**0.303**	**0.302**	**0.303**	**0.409**	**0.409**	**0.410**

Note: Models adjusted for all variables shown in the table, along with fixed effects for each GP practice (N=1,419).

Supplementary Figure S1. Flow diagram of patient study inclusion

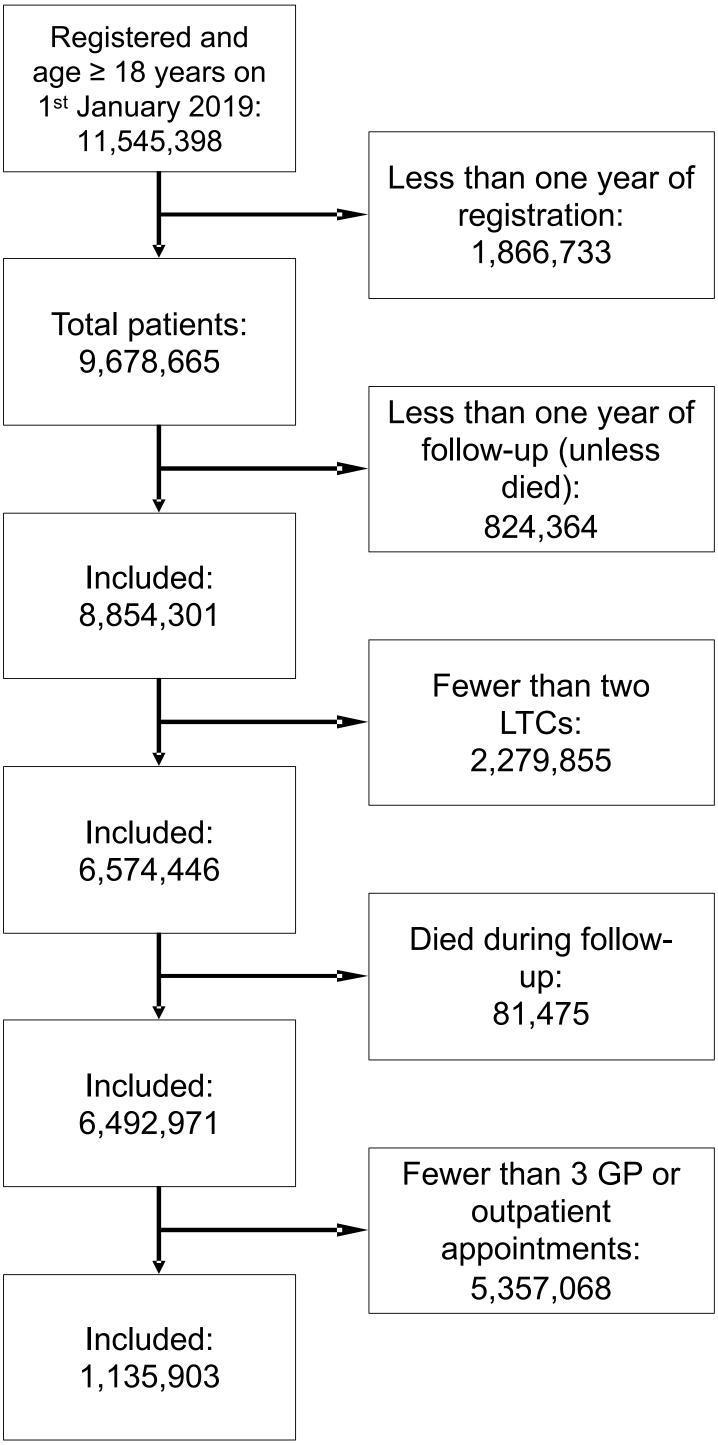

Supplementary Figure S2. Expected GP Continuity of Care Index scores, according to quintile of outpatient Continuity of Care Index scores, in unadjusted, partially adjusted and fully adjusted models

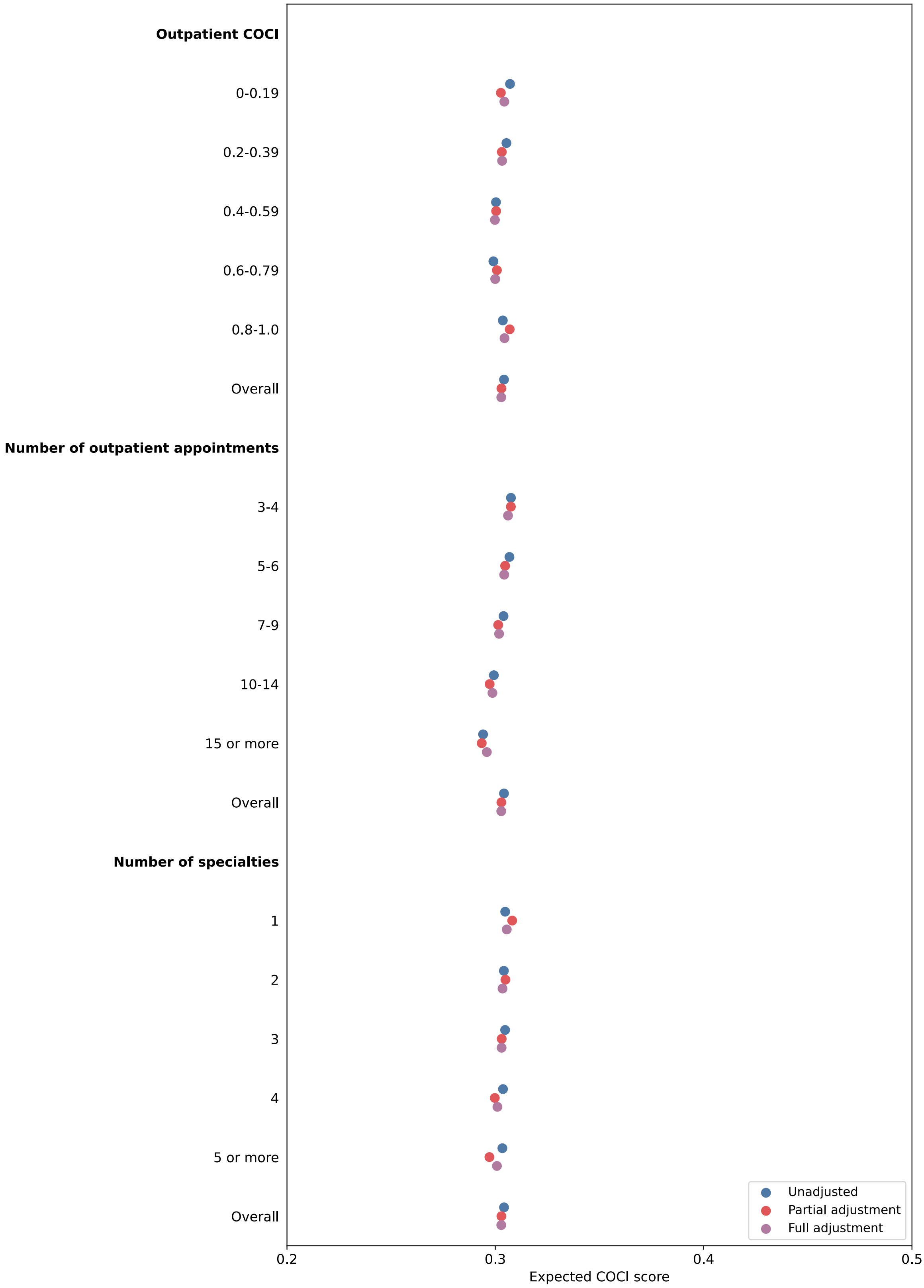

Note: uses same x-axis limits as Figure 1in the main text for comparison of relative impact.

Supplementary Table S4. Expected values of GP Continuity of Care Index (COCI) by total yearly outpatient appointments from univariable, partially adjusted and fully adjusted fractional logit regression models

Outpatient appointments	Univariable			Partially adjusted (sociodemographics)			Fully adjusted (sociodemographics and total number of GP consultations)		
	Coefficient	Lower 95% CI	Upper 95% CI	Coefficient	Lower 95% CI	Upper 95% CI	Coefficient	Lower 95% CI	Upper 95% CI
**3-4**	0.308	0.307	0.308	0.307	0.307	0.308	0.306	0.305	0.307
**5-6**	0.307	0.306	0.308	0.305	0.304	0.306	0.304	0.303	0.305
**7-9**	0.304	0.303	0.305	0.301	0.300	0.303	0.302	0.301	0.303
**10-14**	0.299	0.298	0.301	0.297	0.296	0.299	0.299	0.297	0.300
**15 or more**	0.294	0.293	0.296	0.293	0.292	0.295	0.296	0.294	0.297
**Overall**	0.304	0.304	0.305	0.303	0.302	0.303	0.303	0.302	0.303

Supplementary Table S5. Expected values of GP Continuity of Care Index (COCI) by number of outpatient specialities from univariable, partially adjusted and fully adjusted fractional logit regression models

Number of specialties	Univariable			Partially adjusted (sociodemographics)			Fully adjusted (sociodemographics and total number of GP consultations)		
	Coefficient	Lower 95% CI	Upper 95% CI	Coefficient	Lower 95% CI	Upper 95% CI	Coefficient	Lower 95% CI	Upper 95% CI
**1**	0.305	0.303	0.306	0.308	0.307	0.310	0.305	0.304	0.307
**2**	0.304	0.303	0.305	0.305	0.304	0.306	0.303	0.303	0.304
**3**	0.305	0.304	0.306	0.303	0.302	0.304	0.303	0.302	0.304
**4**	0.304	0.302	0.305	0.300	0.299	0.301	0.301	0.300	0.302
**5 or more**	0.303	0.302	0.305	0.297	0.296	0.298	0.301	0.300	0.302
**Overall**	0.304	0.304	0.305	0.303	0.302	0.303	0.303	0.302	0.303

Supplementary Table S6. Distributions of difference in COCI scores for outpatients and general practice settings per cluster, relative to the population average

Cluster	Median (IQR): outpatient	Median (IQR): GP
Alcohol and liver	-0.086 (-0.093,-0.043)	0.003 (-0.006,0.004)
Allergic, skin and pain	-0.020 (-0.033,-0.009)	-0.003 (-0.007,-0.000)
Autoimmune and coagulopathy	-0.038 (-0.069,-0.032)	-0.004 (-0.006,0.000)
Autoimmune: HLA-B27 linked	-0.023 (-0.044,-0.012)	-0.004 (-0.008,-0.002)
Cardiac: arrhythmia, HF and valve	-0.030 (-0.044,-0.016)	-0.005 (-0.006,-0.003)
GI and anaemias	-0.037 (-0.042,-0.032)	-0.007 (-0.009,0.000)
Haematological and autoimmune	-0.030 (-0.053,-0.015)	-0.005 (-0.011,-0.000)
Lipid and MSK	-0.026 (-0.034,-0.018)	0.000 (-0.004,0.004)
MH and LD	-0.016 (-0.023,-0.006)	0.009 (-0.003,0.014)
Malignancies	-0.015 (-0.039,0.041)	0.006 (-0.004,0.009)
Metabolic	-0.021 (-0.024,-0.015)	-0.002 (-0.004,0.002)
Respiratory and vascular	-0.034 (-0.044,-0.029)	-0.010 (-0.018,-0.008)
Thyroid and other malignancies	-0.040 (-0.067,-0.018)	0.004 (0.004,0.007)
Urinary, neuropathy and female malignancies	-0.033 (-0.037,-0.023)	-0.004 (-0.009,0.003)
Visual, cognitive and bone	-0.023 (-0.029,0.000)	-0.003 (-0.007,-0.001)

Supplementary Figure S3. Expected Continuity of Care Index scores by socio-demographic factors, for general practice versus hospital outpatients in frequent attenders

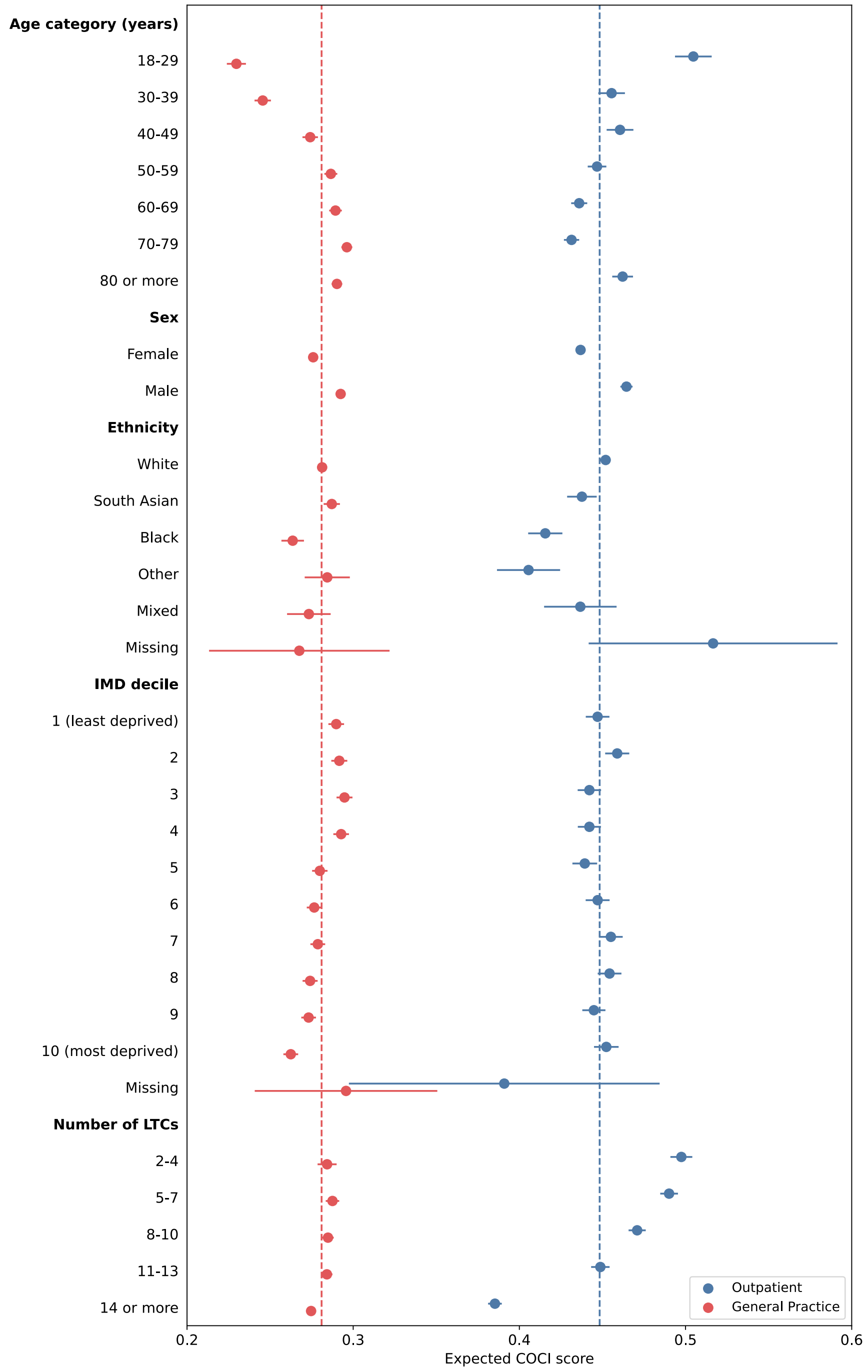

Note: from fractional outcome regression, performed separately for general practice COCI and outpatient COCI, including all covariates in the figure. Unlike the primary analysis, these models do not include number of appointments as the analysis conditions on frequent attendance. Dots represent expected values and bars represent confidence intervals (exact values given in Supplementary Table 6). Dashed lines represent the expected score for the whole population.

Supplementary Table S7. Expected values of GP and outpatient Continuity of Care Index (COCI) for categories of age, sex, ethnicity, deprivation and number of LTCs, from multivariable fractional logit regression models, in frequent attenders

	GP (N=77,283)			Outpatient (N=43,796)		
**Variable**	**Expected COCI**	**Lower 95% CI**	**Upper 95% CI**	**Expected COCI**	**Lower 95% CI**	**Upper 95% CI**
**Age category (years)**						
18-29	0.230	0.224	0.235	0.505	0.494	0.516
30-39	0.246	0.241	0.250	0.455	0.447	0.464
40-49	0.274	0.269	0.279	0.461	0.453	0.469
50-59	0.287	0.283	0.290	0.447	0.441	0.452
60-69	0.289	0.286	0.293	0.436	0.431	0.441
70-79	0.296	0.293	0.299	0.431	0.427	0.436
80 or more	0.290	0.287	0.293	0.462	0.456	0.468
**Sex**						
Female	0.276	0.274	0.278	0.437	0.434	0.440
Male	0.292	0.290	0.295	0.464	0.461	0.468
**Ethnicity**						
White	0.281	0.280	0.283	0.452	0.449	0.454
South Asian	0.287	0.282	0.292	0.438	0.429	0.447
Black	0.264	0.257	0.270	0.416	0.405	0.426
Other	0.284	0.271	0.298	0.406	0.387	0.425
Mixed	0.273	0.260	0.286	0.437	0.415	0.459
Missing	0.268	0.213	0.322	0.517	0.442	0.591
**Index of Multiple Deprivation decile**						
1 (least deprived)	0.290	0.285	0.294	0.447	0.440	0.454
2	0.292	0.287	0.296	0.459	0.452	0.466
3	0.295	0.290	0.300	0.442	0.435	0.449
4	0.293	0.288	0.297	0.442	0.435	0.449
5	0.280	0.275	0.285	0.439	0.432	0.447
6	0.277	0.272	0.281	0.447	0.440	0.454
7	0.279	0.274	0.283	0.455	0.448	0.462
8	0.274	0.270	0.279	0.454	0.447	0.461
9	0.273	0.269	0.278	0.445	0.438	0.452
10 (most deprived)	0.262	0.258	0.267	0.452	0.445	0.460
Missing	0.296	0.241	0.351	0.391	0.297	0.484
**Number of LTCs**						
2-4	0.284	0.279	0.290	0.497	0.491	0.504
5-7	0.288	0.283	0.292	0.490	0.485	0.495
8-10	0.285	0.281	0.288	0.471	0.466	0.476
11-13	0.284	0.281	0.288	0.449	0.443	0.454
14 or more	0.275	0.272	0.277	0.385	0.381	0.389
**Overall**	**0.281**	**0.280**	**0.282**	**0.448**	**0.446**	**0.451**

Note: Models adjusted for all variables shown in the table. Unlike the primary analysis, these models do not include number of appointments as the analysis conditions on frequent attendance.

Supplementary Figure S4. Expected SECON scores by socio-demographic factors, for general practice versus hospital outpatients

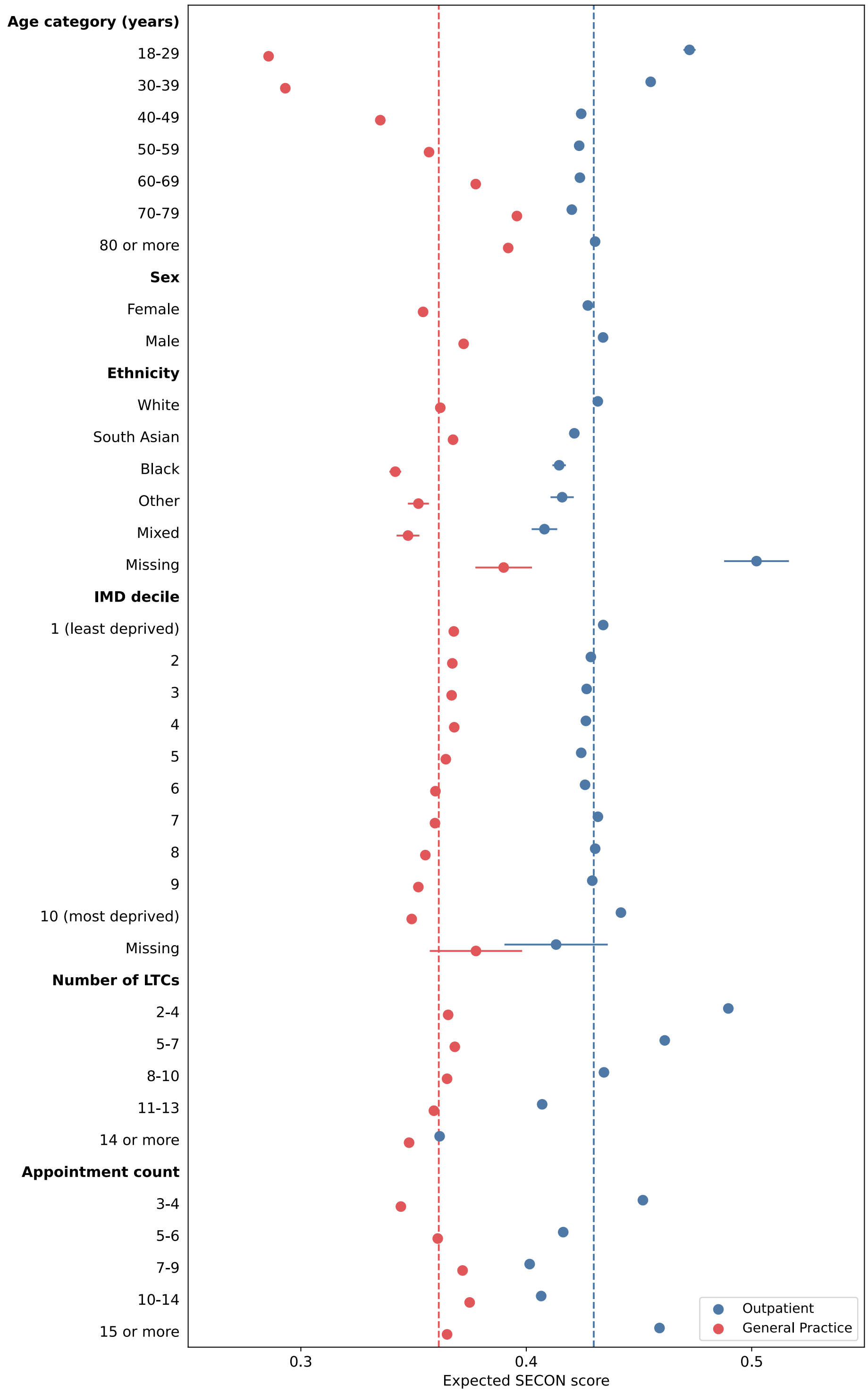

Note: from fractional outcome regression, performed separately for general practice COCI and outpatient COCI, including all covariates in the figure. Point estimates and confidence intervals are given in Supplementary Table 8. Dots represent expected values and bars represent confidence intervals (exact values given in Supplementary Table 7). Dashed lines represent the expected score for the whole population.

Supplementary Table S8. Expected values of GP and outpatient SECON for categories of age, sex, ethnicity, deprivation and number of LTCs, from multivariable fractional logit regression models

	GP			Outpatient		
**Variable**	**Expected COCI**	**Lower 95% CI**	**Upper 95% CI**	**Expected COCI**	**Lower 95% CI**	**Upper 95% CI**
**Age category (years)**						
18-29	0.286	0.284	0.288	0.472	0.470	0.475
30-39	0.293	0.291	0.295	0.455	0.453	0.457
40-49	0.335	0.334	0.337	0.424	0.422	0.426
50-59	0.357	0.356	0.358	0.423	0.422	0.425
60-69	0.378	0.376	0.379	0.424	0.422	0.425
70-79	0.396	0.395	0.397	0.420	0.419	0.422
80 or more	0.392	0.391	0.393	0.431	0.429	0.432
**Sex**						
Female	0.354	0.354	0.355	0.427	0.427	0.428
Male	0.372	0.371	0.373	0.434	0.433	0.435
**Ethnicity**						
White	0.362	0.361	0.362	0.432	0.431	0.432
South Asian	0.368	0.365	0.370	0.421	0.419	0.424
Black	0.342	0.339	0.345	0.415	0.412	0.418
Other	0.352	0.347	0.357	0.416	0.411	0.421
Mixed	0.348	0.342	0.353	0.408	0.402	0.414
Missing	0.390	0.377	0.403	0.502	0.488	0.517
**Index of Multiple Deprivation decile**						
1 (least deprived)	0.368	0.366	0.370	0.434	0.432	0.436
2	0.367	0.366	0.369	0.429	0.427	0.431
3	0.367	0.365	0.369	0.427	0.425	0.429
4	0.368	0.366	0.370	0.426	0.425	0.428
5	0.364	0.363	0.366	0.424	0.422	0.426
6	0.360	0.358	0.361	0.426	0.424	0.428
7	0.359	0.358	0.361	0.432	0.430	0.434
8	0.355	0.354	0.357	0.431	0.429	0.432
9	0.352	0.350	0.354	0.429	0.427	0.431
10 (most deprived)	0.349	0.347	0.351	0.442	0.440	0.444
Missing	0.378	0.357	0.398	0.413	0.390	0.436
**Number of LTCs**						
2-4	0.365	0.364	0.367	0.490	0.488	0.491
5-7	0.368	0.367	0.369	0.461	0.460	0.463
8-10	0.365	0.364	0.366	0.434	0.433	0.436
11-13	0.359	0.358	0.360	0.407	0.406	0.408
14 or more	0.348	0.347	0.349	0.362	0.360	0.363
**Appointment count**						
3-4	0.344	0.343	0.346	0.452	0.451	0.453
5-6	0.361	0.360	0.362	0.416	0.415	0.418
7-9	0.372	0.371	0.373	0.402	0.400	0.403
10-14	0.375	0.374	0.376	0.407	0.405	0.408
15 or more	0.365	0.364	0.366	0.459	0.458	0.461
**Overall**	**0.361**	**0.361**	**0.362**	**0.430**	**0.429**	**0.431**

Note: Models adjusted for all variables shown in the table.

Supplementary Table S9. Expected values of GP SECON by quintiles of outpatient specialty SECON from univariable, partially adjusted and fully adjusted fractional logit regression models

Outpatient SECON	Univariable			Partially adjusted (sociodemographics)			Fully adjusted (sociodemographics and total number of GP consultations)		
	Coefficient	Lower 95% CI	Upper 95% CI	Coefficient	Lower 95% CI	Upper 95% CI	Coefficient	Lower 95% CI	Upper 95% CI
**0-0.19**	0.371	0.370	0.372	0.367	0.366	0.368	0.366	0.365	0.367
**0.2-0.39**	0.364	0.363	0.365	0.361	0.360	0.362	0.360	0.359	0.361
**0.4-0.59**	0.360	0.359	0.361	0.360	0.359	0.361	0.360	0.359	0.361
**0.6-0.79**	0.356	0.355	0.358	0.357	0.356	0.359	0.357	0.356	0.359
**0.8-1.0**	0.353	0.351	0.354	0.357	0.356	0.359	0.360	0.358	0.361
**Overall**	0.362	0.362	0.363	0.361	0.361	0.362	0.361	0.361	0.362

Supplementary Table S10. Expected difference in COCI for those with each disease compared with those without, in outpatients and General Practice

Disease	Expected difference in COCI for outpatients	Expected difference in COCI for GP	Cluster
Abdominal Aortic Aneurysm	-0.058	-0.01	Respiratory and vascular
Abdominal Hernia	-0.023	-0.007	Respiratory and vascular
Acne	0.001	-0.008	Allergic, skin and pain
Alcoholic liver disease	-0.056	0.002	Alcohol and liver
Alcohol Misuse	-0.001	-0.005	Alcohol and liver
Allergic and chronic rhinitis	-0.011	-0.006	Allergic, skin and pain
Alopecia areata	-0.004	-0.001	Allergic, skin and pain
Anaemia_other	-0.031	-0.009	GI and anaemias
Angiodysplasia of colon	-0.054	0	GI and anaemias
Ankylosing spondylitis	-0.049	-0.001	Autoimmune: HLA-B27 linked
Anterior and Intermediate Uveitis	-0.01	-0.003	Autoimmune: HLA-B27 linked
Anxiety disorders	-0.016	-0.001	MH and LD
Aplastic anaemias	-0.02	-0.016	Haematological and autoimmune
Asbestosis	-0.052	-0.029	Respiratory and vascular
Asthma	-0.019	-0.011	Allergic, skin and pain
Atrial Fibrillation	-0.017	-0.004	Cardiac: arrhythmia, HF and valve
Atrioventricular blocks	-0.039	0	Cardiac: arrhythmia, HF and valve
Autism and Asperger's syndrome	-0.004	0.035	MH and LD
Autoimmune liver disease	-0.09	0.003	Alcohol and liver
Autonomic Neuropathy	-0.053	0.015	Metabolic
Barrett's oesophagus	-0.044	-0.01	GI and anaemias
Benign essential tremor	-0.036	0.005	Lipid and MSK
Benign Prostatic Hyperplasia	-0.029	-0.01	Respiratory and vascular
Bipolar affective disorder and mania	0.006	0.01	MH and LD
Bronchiectasis	-0.055	-0.017	Haematological and autoimmune
Cardiomyopathy_other	-0.005	-0.005	Cardiac: arrhythmia, HF and valve
Cataract	-0.012	-0.005	Visual, cognitive and bone
Cerebral Palsy	-0.039	0.008	MH and LD
Chronic Cystitis	-0.033	-0.01	Urinary, neuropathy and female malignancies
Chronic Fatigue Syndrome	-0.047	0.013	Allergic, skin and pain
Chronic Kidney Disease	-0.015	-0.002	Metabolic
Chronic primary pain	-0.067	0.022	Allergic, skin and pain
Chronic sinusitis	-0.021	-0.011	Allergic, skin and pain
Chronic viral hepatitis	-0.028	-0.008	Alcohol and liver
Cirrhosis	-0.086	0.004	Alcohol and liver
Coeliac disease	-0.056	0	GI and anaemias

Collapsed vertebra	-0.034	-0.007	Visual, cognitive and bone
Congenital Septal Defect	-0.051	-0.007	Cardiac: arrhythmia, HF and valve
COPD	-0.03	-0.023	Respiratory and vascular
Coronary Heart Disease (not otherwise specified)	-0.029	-0.009	Respiratory and vascular
Crohn's disease	-0.028	-0.007	Autoimmune: HLA-B27 linked
Cystic Fibrosis	0.013	0.024	MH and LD
Dementia	0.012	-0.024	Visual, cognitive and bone
Depression	-0.021	0.003	MH and LD
Dermatitis	-0.006	-0.007	Allergic, skin and pain
Diabetes Mellitus_other or not specified	-0.023	-0.003	Metabolic
Diabetic Eye Disease	-0.023	-0.004	Metabolic
Diabetic Neuropathy	-0.047	-0.008	Metabolic
Diaphragmatic hernia	-0.041	-0.007	GI and anaemias
Dilated cardiomyopathy	0.017	-0.002	Cardiac: arrhythmia, HF and valve
Diverticular Disease	-0.037	-0.007	GI and anaemias
Down's syndrome	-0.031	-0.006	MH and LD
Dysmenorrhoea	-0.014	-0.006	Allergic, skin and pain
Eating Disorders	-0.018	0.013	MH and LD
Endometrial hyperplasia and hypertrophy	-0.019	-0.007	Urinary, neuropathy and female malignancies
Endometriosis	-0.034	-0.004	Allergic, skin and pain
Enteropathic arthropathy	-0.095	-0.017	Autoimmune: HLA-B27 linked
Enthesopathy and synovial disorder	-0.019	-0.008	Lipid and MSK
Epilepsy	-0.038	-0.005	MH and LD
Fatty Liver	-0.046	-0.008	Alcohol and liver
Fibromatosis	-0.016	-0.003	Lipid and MSK
Folate deficiency anaemia	-0.037	0.01	GI and anaemias
Gastritis and duodenitis	-0.039	-0.009	GI and anaemias
Gastro-oesophageal reflux disease	-0.033	-0.007	GI and anaemias
Giant Cell arteritis	-0.051	0.003	Visual, cognitive and bone
Glaucoma	-0.013	0.001	Visual, cognitive and bone
Glomerulonephritis	-0.003	-0.013	Autoimmune and coagulopathy
Gout	-0.015	-0.003	Metabolic
Hearing loss	-0.026	-0.012	Lipid and MSK
Heart failure	-0.02	-0.003	Cardiac: arrhythmia, HF and valve
Hepatic failure	-0.109	0.006	Alcohol and liver
Hidradenitis suppurativa	-0.03	-0.002	Allergic, skin and pain
HIV	-0.017	-0.005	Haematological and autoimmune
Hodgkin Lymphoma	-0.046	0.015	Haematological and autoimmune
Hyperkinetic disorders	-0.013	0.014	MH and LD
Hyperparathyroidism	-0.049	0.002	Autoimmune and coagulopathy

Hypersplenism	-0.056	-0.001	Haematological and autoimmune
Hypertension	-0.015	0.01	Metabolic
Hypertrophic Cardiomyopathy	-0.025	-0.008	Cardiac: arrhythmia, HF and valve
Hypertrophic Nasal Turbinates	-0.036	0.002	Allergic, skin and pain
Hyposplenism	-0.037	0.001	Haematological and autoimmune
Idiopathic Intracranial Hypertension	-0.106	0.007	Allergic, skin and pain
Immunodeficiencies	-0.096	0.002	Haematological and autoimmune
Intellectual disability	-0.008	-0.018	MH and LD
Intervertebral disc disorders	-0.037	0	Lipid and MSK
Intracerebral haemorrhage	-0.024	0.003	Visual, cognitive and bone
Irritable bowel syndrome	-0.03	-0.003	Allergic, skin and pain
Ischaemic stroke	-0.028	-0.002	Visual, cognitive and bone
Left bundle branch block	-0.012	-0.003	Cardiac: arrhythmia, HF and valve
Leukaemia	0	-0.009	Haematological and autoimmune
Low HDL-C	-0.02	0.003	Metabolic
Lupus Erythematosus	-0.082	-0.005	Autoimmune and coagulopathy
Macular degeneration	0.049	-0.007	Visual, cognitive and bone
Meniere's Disease	-0.032	0	Lipid and MSK
Migraine	-0.028	0	Allergic, skin and pain
Motor neurone disease	-0.02	0	Haematological and autoimmune
Multiple sclerosis	-0.019	0	Urinary, neuropathy and female malignancies
Multiple valve disorder	-0.038	-0.006	Cardiac: arrhythmia, HF and valve
Myasthenia gravis	-0.076	0.009	Haematological and autoimmune
Myelodysplastic Syndrome	0.017	-0.012	Haematological and autoimmune
Myocardial Infarction	-0.023	-0.009	Respiratory and vascular
Neuropathic Bladder	-0.04	-0.004	Urinary, neuropathy and female malignancies
Non-Hodgkin Lymphoma	-0.028	-0.005	Haematological and autoimmune
Nonrheumatic aortic valve disorders	-0.05	-0.003	Cardiac: arrhythmia, HF and valve
Nonrheumatic mitral valve disorders	-0.027	-0.006	Cardiac: arrhythmia, HF and valve
Obesity	-0.016	0.002	Metabolic
Obsessive-compulsive disorder	-0.012	0.013	MH and LD
Obstructive and reflux uropathy	-0.036	-0.014	Urinary, neuropathy and female malignancies
Oesophageal varices	-0.097	0.003	Alcohol and liver
Osteoarthritis (excl spine)	-0.024	-0.004	Lipid and MSK
Osteoporosis	-0.033	-0.002	Visual, cognitive and bone
Other haemolytic anaemias	-0.015	-0.009	Haematological and autoimmune
Pancreatitis	-0.041	-0.007	Alcohol and liver
Parkinson's disease	-0.047	0	Lipid and MSK
Pericardial Effusion	-0.048	-0.004	Cardiac: arrhythmia, HF and valve
Peripheral Neuropathy	-0.039	-0.004	Metabolic

Peripheral Vascular Disease	-0.039	-0.011	Respiratory and vascular
Personality disorders	-0.026	0.019	MH and LD
Plasma Cell Malignancy	0.069	-0.013	Haematological and autoimmune
Pleural effusion	-0.031	-0.005	Cardiac: arrhythmia, HF and valve
Pleural plaque	-0.041	-0.023	Respiratory and vascular
Polycystic ovarian syndrome	-0.017	-0.001	Allergic, skin and pain
Polycythaemia vera	0.04	0	Visual, cognitive and bone
Polymyalgia Rheumatica	-0.026	0.017	Lipid and MSK
Portal hypertension	-0.112	0.004	Alcohol and liver
Posterior Uveitis	0.003	-0.003	Autoimmune: HLA-B27 linked
Primary Malignancy_Biliary Tract	0.012	0.011	Malignancies
Primary Malignancy_Bladder	-0.031	-0.013	GI and anaemias
Primary Malignancy_Bone	-0.06	0.008	Malignancies
Primary Malignancy_Bowel	-0.039	0.006	Malignancies
Primary Malignancy_Brain	-0.067	0.027	Thyroid and other malignancies
Primary Malignancy_Breast	-0.047	0.008	Urinary, neuropathy and female malignancies
Primary Malignancy_Cervix	-0.029	0.001	Urinary, neuropathy and female malignancies
Primary Malignancy_Kidney	-0.057	-0.006	Malignancies
Primary Malignancy_Liver	-0.088	0.015	Alcohol and liver
Primary Malignancy_Lung	-0.015	-0.011	Malignancies
Primary Malignancy_Melanoma	-0.018	0.002	Thyroid and other malignancies
Primary Malignancy_Oesophageal	-0.039	0.011	Malignancies
Primary Malignancy_Oropharyngeal	-0.044	-0.004	Malignancies
Primary Malignancy_other	-0.036	-0.008	Malignancies
Primary Malignancy_Ovary	0.01	0.009	Urinary, neuropathy and female malignancies
Primary Malignancy_Pancreas	0.009	0.014	Malignancies
Primary Malignancy_Prostate	-0.025	0.004	Malignancies
Primary Malignancy_Skin	-0.018	-0.003	Lipid and MSK
Primary Malignancy_Stomach	-0.027	-0.001	Malignancies
Primary Malignancy_Testis	-0.04	0.007	Thyroid and other malignancies
Primary Malignancy_Thyroid	-0.104	0.004	Thyroid and other malignancies
Primary Malignancy_Uterus	-0.025	0.011	Urinary, neuropathy and female malignancies
Primary pulmonary hypertension	-0.046	-0.012	Cardiac: arrhythmia, HF and valve
Primary Thrombocytopaenia	-0.007	-0.012	Haematological and autoimmune
Psoriasis	0.001	-0.005	Autoimmune: HLA-B27 linked
Psoriatic Arthritis	-0.019	-0.002	Autoimmune: HLA-B27 linked
Pulmonary Fibrosis	-0.056	-0.013	Autoimmune and coagulopathy
Raised LDL-C	-0.005	0.004	Lipid and MSK
Raised Total Cholesterol	-0.007	0.012	Lipid and MSK

Raised Triglycerides	-0.015	0.002	Metabolic
Raynaud's syndrome	-0.035	0.001	Autoimmune and coagulopathy
Retinal vascular occlusions	0.031	-0.003	Visual, cognitive and bone
Rheumatic Valve Disorder	-0.014	-0.003	Cardiac: arrhythmia, HF and valve
Rheumatoid Arthritis	-0.036	-0.006	Autoimmune and coagulopathy
Right bundle branch block combinations	-0.03	-0.014	Cardiac: arrhythmia, HF and valve
Rosacea	-0.009	-0.005	Lipid and MSK
Sarcoidosis	-0.055	0.002	Autoimmune: HLA-B27 linked
Schizophrenia	0.036	-0.004	MH and LD
Scleritis and episcleritis	-0.017	-0.009	Autoimmune: HLA-B27 linked
Scleroderma	-0.124	-0.001	Autoimmune and coagulopathy
Seborrheic dermatitis	-0.009	-0.007	Allergic, skin and pain
Secondary Malignancy_Bone	0.067	-0.009	Malignancies
Secondary Malignancy_Brain	0.061	0.018	Malignancies
Secondary Malignancy_Liver	0.046	0.007	Malignancies
Secondary Malignancy_Lung	0.041	0.006	Malignancies
Secondary Malignancy_Lymph Nodes	-0.009	0.001	Malignancies
Secondary Malignancy_other	0.049	0.009	Malignancies
Secondary polycythaemia	-0.018	-0.003	Respiratory and vascular
Secondary pulmonary hypertension	-0.043	-0.005	Cardiac: arrhythmia, HF and valve
Secondary Thrombocytopaenia	-0.033	-0.003	Haematological and autoimmune
Sickle Cell Disease	-0.078	-0.003	Haematological and autoimmune
Sick sinus syndrome	-0.052	0.006	Cardiac: arrhythmia, HF and valve
Sjogren's Syndrome	-0.092	-0.002	Autoimmune and coagulopathy
Sleep apnoea	-0.082	-0.001	Respiratory and vascular
Spina bifida	-0.043	-0.009	Urinary, neuropathy and female malignancies
Spinal stenosis	-0.051	0.003	Lipid and MSK
Spondylolisthesis	-0.034	0.007	Lipid and MSK
Spondylosis	-0.03	-0.001	Lipid and MSK
Stable Angina	-0.034	-0.008	Respiratory and vascular
Stroke_not otherwise specified	-0.023	-0.01	Visual, cognitive and bone
Subarachnoid haemorrhage	-0.024	-0.001	Visual, cognitive and bone
Substance Misuse	-0.016	0.009	MH and LD
Supraventricular tachycardia	-0.032	-0.005	Cardiac: arrhythmia, HF and valve
Thalassaemia	-0.04	-0.006	Haematological and autoimmune
Thrombophilia	-0.038	-0.004	Autoimmune and coagulopathy
Thyroid Disease	-0.018	0.004	Thyroid and other malignancies
Tinnitus	-0.024	-0.005	Lipid and MSK
Transient ischaemic attack	-0.031	-0.007	Visual, cognitive and bone

Trigeminal neuralgia	-0.034	-0.003	Urinary, neuropathy and female malignancies
Tubulo-interstitial nephritis	-0.006	0.028	Autoimmune and coagulopathy
Type 1 Diabetes Mellitus	-0.021	-0.004	Metabolic
Type 2 Diabetes Mellitus	-0.024	-0.002	Metabolic
Ulcerative colitis	-0.03	-0.008	Autoimmune: HLA-B27 linked
Unstable Angina	-0.044	-0.018	Respiratory and vascular
Urinary Incontinence	-0.036	-0.011	Urinary, neuropathy and female malignancies
Venous thromboembolic disease (Excl PE)	-0.029	-0.006	Autoimmune and coagulopathy
Ventricular tachycardia	0.002	-0.006	Cardiac: arrhythmia, HF and valve
Visual impairment and blindness	-0.016	-0.009	Visual, cognitive and bone
Vitamin B12 deficiency anaemia	-0.03	0.008	GI and anaemias
Vitiligo	0.001	-0.002	Allergic, skin and pain

References

1. Davidson, J. *et al.* Codelists for: ‘Ethnic differences in the incidence of clinically diagnosed influenza: an England population-based cohort study 2008-2018’. https://datacompass.lshtm.ac.uk/id/eprint/2102/ (2021) doi:10.17037/DATA.00002102.
